# Changing parental perceptions to childhood immunisations during the COVID-19 pandemic in the UK: evidence from three cross-sectional surveys between 2020 and 2022

**DOI:** 10.1136/bmjph-2024-002536

**Published:** 2025-11-27

**Authors:** Rosie Thistlethwayte, Alexandre de Figueiredo

**Affiliations:** 1 Department of Infectious Disease Epidemiology, London School of Hygiene & Tropical Medicine, London, UK; 2 Laboratory of Data Discovery for Health Limited (D24H), Hong Kong Special Administrative Region, People's Republic of China

**Keywords:** COVID-19, Vaccination, Public Health

## Abstract

**Introduction:**

Childhood immunisation rates in the UK have recently fallen to their lowest level in 14 years. However, there is limited temporal evidence on how parental attitudes have evolved during the COVID-19 pandemic, restricting our understanding of its broader impact. This study explores trends in parental confidence in childhood immunisations between 2020 and 2022 across the UK and how socio-demographic predictors of vaccine perceptions have shifted over time.

**Methods:**

This study draws on data from three nationally representative, cross-sectional surveys conducted in 2020, 2021 and 2022 including 14 720 adults responsible for decisions on childhood vaccination. Multiple logistic regression assesses changes in reported childhood vaccine refusal over time, by region, socio-demographic group and vaccine. Additional 2022 analysis explores how parental perceptions of childhood immunisations have changed since the start of the pandemic.

**Results:**

UK-wide refusal decreased from 17.3% (16.4 to 18.5%) in 2020 to 14.8% (13.7 to 15.7%) in 2021 before increasing to 21.1% (19.8 to 22.5%) in 2022, largely due to high refusal rates for the COVID-19 vaccine for children. Parents who had not received at least three COVID-19 vaccine doses were more likely to report declining confidence in childhood vaccines, suggesting a spillover of COVID-19 vaccine hesitancy onto routine vaccines. Refusal was higher among younger respondents, London residents, Hindus and Muslims. Interestingly, however, 18–34-year-olds, Hindus, Muslims and Black/Black British respondents reported becoming more positive towards childhood vaccines in 2022 compared with 2020.

**Conclusions:**

Parental refusal of routine childhood immunisations decreased between 2020 and 2022 and remains low across the UK. Encouragingly, groups with historically lower confidence reported more positive attitudes in 2022. However, this has not been matched by increased uptake. Parents who received fewer than three COVID-19 doses are more likely to have become less positive over time and represent a key group for targeted outreach to rebuild confidence and support uptake.

**WHAT IS ALREADY KNOWN ON THIS TOPIC**

**WHAT THIS STUDY ADDS**

- This study quantifies the change in parental refusal of routine childhood vaccines between 2020 and 2022 and examines the demographic and economic factors influencing vaccination intent.
- Although overall refusal of childhood vaccine initially declined between 2020 and 2021, it rose sharply between 2021 and 2022.
- Encouragingly, groups with historically lower vaccine confidence—such as younger adults, ethnic minorities and religious minorities—reported more positive attitudes in 2022 compared with 2020.

**HOW THIS STUDY MIGHT AFFECT RESEARCH, PRACTICE OR POLICY**

- This study identifies parents with lower COVID-19 vaccine uptake as a key group for targeted interventions to prevent spillover of hesitancy towards routine childhood vaccines.
- The finding that attitudes towards childhood vaccines have become more positive in some groups who were previously more hesitant supports continued investment in targeted outreach and engagement with these groups.

## Introduction

 Since the start of the COVID-19 pandemic, declining childhood vaccination rates^1 2^ have raised concerns about the extent to which the reduced uptake is due to falling vaccine confidence or pandemic-induced practical barriers. Data from the UK Health Security Agency (UKHSA) show that routine childhood vaccine uptake in the UK remains below pre-pandemic levels,^3^ with measles coverage particularly alarming, as it continues to fall short of the WHO’s 95% target for elimination.^4 5^ The UKHSA’s June 2024 vaccine coverage update^6^ highlights further declines in vaccines administered before 24 months of age, with the measles, mumps and rubella (MMR) vaccine coverage lagging behind other routine immunisations. MMR coverage in England has steadily decreased over the past decade, with second-dose MMR (MMR2) coverage dropping from 88.6% in 2014–2015^7^ to just under 85% by late 2023.^6^

Of notable concern in the UK is the significant geographic variation in vaccine uptake, especially in regions like London, North West England and the East Midlands.^1^ In Hackney, MMR2 coverage is as low as 60.4%, and only five of 33 local authorities in London have achieved coverage above 80%.^1^ Disruptions to healthcare services,^8^ pandemic restrictions^9^ and the reallocation of resources^10^ have been cited as reasons for declining routine immunisation rates during the pandemic. During the first national lockdown, a survey of 1252 parents by Bell *et al* found that fear of catching COVID-19 and confusion over the availability of vaccination services were key factors driving low vaccine uptake.^11^ Although catch-up campaigns are underway,^12^ routine immunisation coverage among children remains lower than pre-pandemic levels.^13^

The impact of the pandemic on demand for childhood vaccines (rather than practical barriers) remains poorly understood. While the decline in routine immunisations in the UK and globally is well-documented, there is limited evidence on how the pandemic influenced demand for vaccines other than the COVID-19 vaccine. Several factors suggest that the pandemic may have affected broader vaccine hesitancy. Increased public awareness of immunity, disease transmission and vaccine development might have boosted parental confidence in vaccines. However, vaccine fatigue,^14^ misinformation^15^ and potential backfire effects—such as psychological reactance^16^ to vaccine certification policies in the UK^17 18^ and elsewhere^19 20^—may have contributed to COVID-19 vaccine hesitancy, with possible spillover effects on other vaccines.^21^ Precedents for such spillover exist: after the 2017 Dengvaxia controversy in the Philippines, vaccine confidence dropped from 82% in 2015 to just 22% in 2018,^22^ leading to a substantial decline in routine childhood immunisations. MMR vaccine uptake, for example, fell by over 30 percentage points, while measles cases surged by 2000% compared with 2017.^23^

A handful of studies have examined changes in attitudes toward childhood vaccines during the COVID-19 pandemic, most of which have found declines in vaccine confidence, though the evidence in the UK is limited. In the USA, research indicates decreased vaccine confidence in specific populations,^24 25^ with Shah et al. reporting that from 2020 to 2022, more parents believed childhood vaccines could cause illness, death or harmful side effects in a longitudinal, nationally representative study.^26^ Similarly, declines in vaccine confidence have been observed in Italy^27^ and Turkey.^28^ However, Higgins *et al* found no overall change in parental vaccine hesitancy during the pandemic in the USA,^29^ while data from Finland suggested a positive spillover effect, with more adults viewing vaccines favourably.^30^ At a global scale, UNICEF’s *State of the World’s Children 2023* report highlights a drop in vaccine safety perceptions, especially among younger people.^31^ However, as with other surveys using a vaccine-agnostic confidence scale, it remains unclear whether this decline reflects a genuine drop in confidence across all vaccines or if it is driven primarily by attitudes toward the COVID-19 vaccine. In a global study involving over 23 000 respondents from 23 countries, Lazarus *et al* found that 23.1% of respondents were less willing to get vaccinated for diseases other than COVID-19, while 60.8% were more willing.^21^ In the UK, a UKHSA survey of 1000 parents found that 27% felt ‘more positive about routine childhood vaccines’ after the COVID-19 vaccination campaign, while 12% felt less positive.^32^ However, such findings should be interpreted with caution, as asking about changes in attitude may lead to “response substitution”, where participants report their current attitude rather than an actual change.^33^ A 2024 pre-print by de Figueiredo *et al* also noted a decline in the intent to receive seasonal influenza vaccines among the UK adult population, particularly in some Asian communities.^34^

The impact of the pandemic on attitudes towards childhood vaccines in the UK remains unclear. This study aims to quantify changes in parental intent regarding childhood vaccinations in the UK during the pandemic using three cross-sectional surveys conducted in 2020, 2021 and 2022. Rather than relying on confidence scales—which may be influenced by the introduction of the COVID-19 vaccine—or asking about changes in attitude, this study focuses on cross-sectional measures of vaccine refusal. This study also examines the demographic and socioeconomic factors influencing vaccination intent and how these have evolved since before the pandemic. Understanding these shifts will provide insights into the pandemic’s impact on national and sub-national demand for childhood vaccines.

## Methods

### Data

This study uses data from three cross-sectional online surveys of UK adults, conducted by ORB International (www.orb-international.com). The first was conducted in September and October 2020, before the COVID-19 vaccine rollout, followed by surveys in October 2021 and July-August 2022. In each survey, panel quotas were set based on UK national distributions of age, sex and region to ensure a nationally representative sample. The 2020 and 2021 surveys examined vaccination beliefs among UK adults (aged 18 and over), each with about 17 000 respondents. The 2022 survey focused exclusively on individuals responsible for making vaccination decisions for children. In the first two surveys, this responsibility was identified through the question: “Are you responsible for decisions relating to the vaccination of children?” Respondents who answered affirmatively were included in the final sample, along with all respondents from the 2022 survey. The total sample size across all surveys is 14 280, with 5288 respondents in 2020, 4994 in 2021 and 3988 in 2022.

Three response variables (RVs) are used to measure parental or caregiver attitudes toward childhood immunisations. All respondents are asked, “Are there any vaccines you have not, or would not, allow your child(ren) to have?” (RV1), with three response options: ‘yes’, ‘no’ and ‘do not know’. Respondents who did not answer ‘no’ were then asked, “Which vaccines, if any, do you have concerns about?” (RV2), where they could select one or more of the following options: ‘measles, mumps and rubella (MMR) combined vaccine’, ‘influenza vaccine’, ‘human papillomavirus (HPV) vaccine’ and ‘other (please specify)’. In the 2021 and 2022 surveys, ‘COVID-19 vaccine’ was added to this list, reflecting the rollout of COVID-19 vaccines for children.

In the 2022 survey, an additional question was introduced to explore changes in parental or caregiver attitudes toward vaccination: “Since the beginning of the COVID-19 pandemic, has your attitude towards giving your child(ren) routine vaccines changed? (The COVID-19 vaccine is not considered a routine vaccine)”. Respondents answered on a three-point scale: ‘yes’, ‘no’ or ‘do not know’. Those who selected ‘yes’ were then asked, “How have your attitudes towards other vaccines for your child(ren) changed?” and provided with a four-point scale ranging from “I feel much more positive about giving my child vaccines” to “I feel much less positive about giving my child vaccines”. These two questions were combined to create RV3, which consolidates responses into three categories: ‘more positive’, ‘no change’ and ‘less positive’. This two-step questioning approach may help mitigate issues with asking directly about changes in sentiment over time.^35^

Individuals’ sex, age, ethnicity, religious affiliation, employment status, income, work status, first language and region of residence in the UK are also collected and used as explanatory variables to identify the socioeconomic predictors of the response variables. The first level of the Nomenclature of Territorial Unit for Statistics (NUTS1)^36^ is used for regional classification. The respondent’s own COVID-19 vaccine status was also included in the third survey and is used to explore how attitudes vary according to COVID-19 vaccination status. All study variables (response and predictors) are provided in table 1. Online supplemental table 1 shows the number and percentage of each socio-demographic response variable recorded across all three surveys. Each of the three questionnaires is also provided in the supplementary materials (SM (SM Questionnaire 2020, SM Questionnaire 2021, SM Questionnaire 2022).

**Table 1:** Study response and predictor variables. All responses (RV1–3) and predictor variables are outlined along with variable value options and recodes, as well as the baseline group used for the logistic regression models (see Methods).

Variable type	Survey question	Values	Baseline
RV1	Are there any immunisations you have not, or would not, allow children to have?	Yes, no and do not know (no)	n/a (response variable)
RV2	Which vaccines, if any, do you have concerns about? (Select all that apply)	MMR, HPV, influenza, COVID-19 (2021 and 2022 only), other (please specify) and none of the above.	n/a (response variable)
**RV3** **(2022 only)**	Since the beginning of the COVID-19 pandemic, have your attitudes towards giving your child(ren) routine vaccines changed?	Responses to “Since the beginning of the COVID-19 pandemic, has your attitude towards giving your child(ren) routine vaccines changed? (The COVID-19 vaccine is not considered a routine vaccine)” and “How have your attitudes towards other vaccines for your child(ren) changed?” recoded to ‘more positive’, ‘no change’ and ‘less positive’ (see online supplemental material Questionnaire 2022).	n/a (response variable)
Sex	I am…	Male, female and other (removed from analysis due to low sample size)	Female
Religion	Do you consider yourself…	Atheist/agnostic, Christian, Buddhist (recoded to ‘other’), Hindu, Muslim, Jewish (recoded to ‘other’), other and do not wish to answer.	Christian
Education	What is the highest level of education you have completed? (Select the response that best applies)	0–4 GCSEs, O-levels, or equivalent (recoded to ‘level 1–3’); 2+A levels or equivalents (recoded to ‘level 1–3’); 5+GCSEs, O-levels, 1 A level, or equivalents (recoded to ‘level 1–3’); undergraduate postgraduate degree or other professional qualification (recoded to ‘level 4’); apprenticeship (recoded to ‘none/other’); do not know (recoded to ‘none/other’); do not wish to answer; and other (eg, vocational, foreign qualifications) (recoded to ‘none/other’)	Level 1–3
Age	How old are you?	Integer categorised into 18–24, 25–34, 35–44, 45–54 or 55+	35–44
Region	Which UK region do you live in?	East Midlands, East of England, Greater London, North West, North West, Northern Ireland, Scotland, South East, South West, Wales, West Midlands, Yorkshire and the Humber and other (removed from data)	South East
Ethnicity	Which best describes your ethnicity? (Select the response that best applies)	Asian or Asian British: Chinese (recoded to ‘Asian/Asian British’); Asian or Asian British: Indian (recoded to ‘Asian/Asian British’); Asian or Asian British: Other (recoded to ‘Asian/Asian British’); Asian or Asian British: Pakistani (recoded to ‘Asian/Asian British’); Black, African, Caribbean, or Black British (recoded to ‘Black/Black British’); White and Asian or White and Asian British (recoded to ‘Mixed’); White and Black African (recoded to ‘Mixed’); White and Black Caribbean (recoded to ‘Mixed’); White: English/Welsh/Scottish/Northern Irish/British (recoded to ‘White’); White: Irish (recoded to ‘White’); White: other white background (recoded to ‘White’); Gypsy or Irish traveller (recoded to ‘other’); Roma (recoded to ‘other’); other; and do not wish to answer.	White
Income	What is your total household income in GBP (£) from all sources before tax?	Under £15 000; £15 000–£24 999; £25 000–£34 999; £35 000–£44 999; £45 000–£54 999; £55 000–£64 000; £65 000–£99 999; over £100 000; and do not wish to answer	£25 000–£34 999
Work status	Which of the following best describes your work status 6 months ago?	Working full-time (including self-employed); working part-time (including self-employed); unemployed; student; looking after the home; unable to work (eg, short-term or long-term disability) (recoded to ‘retired/disabled’); retired (recoded to ‘retired/disabled’); and do not wish to answer.	Working full-time
Language	What is your first language?	English or Welsh; Polish (recoded to ‘other’); Punjabi (recoded to ‘other’); Punjabi (recoded to ‘other’); Urdu (recoded to ‘other’); Bengali (recoded to ‘other’); other; and do not wish to answer.	English or Welsh
COVID-19 vaccine status (2022 only)	Which of the following best describes how many COVID-19 vaccines you have currently received?	None, one, two and at least three doses	Two plus a booster

HPV, human papillomavirus; MMR, measles, mumps and rubella; n/a, not applicable; SM, supplementary materials.

### Time-varying trends in vaccine refusal

Binomial logistic regressions are used to obtain the predicted probability (converted to population percentages) of vaccine refusal (RV1) for each respondent in the combined dataset. To generate uncertainty around national and regional refusal rate estimates, a total of 100 bootstrap samples of these probabilities are generated. National and regional level refusal rate estimates (and corresponding CIs) are then obtained by multiplying bootstrapped individual refusal probabilities by inverse proportional weights (IPWs) and summing over all individuals in each survey year. In the absence of population-level census data for adults responsible for vaccinating children—data that would allow for post-stratification—we use IPWs to internally match socio-demographic characteristics across the three survey waves. This method helps ensure consistent demographic representation across samples, minimising inclusion bias and enabling internally valid comparisons over time. In addition to these bootstrapped time-varying estimates of refusal, ORs of association (with baselines specified in table 1) between RV1 and predictor variables are obtained. The same approach as described above is used to generate time-varying estimates and CIs for each of the individual vaccines in RV2 and to generate national estimates and socio-demographic determinants. No evidence of strong multicollinearity was found for the socio-demographic predictors in any model as per the adjusted generalised SE inflation factor (aGSIF),^37^ with all aGSIFs smaller than 1.6. To account for that, the question used to form RV1 (“Are there any immunisations you have not, or would not, allow children to have?”) involves the recode of ‘Don’t know’ to ‘No’ in the main analysis, and a sensitivity analysis where these uncertain responses are excluded from the analysis is also conducted.

### Self-reported changes in attitudes to childhood vaccines

A proportional odds ordinal logistic regression^38^ is used to determine the socio-demographic predictors of self-reported change in attitudes towards childhood routine immunisations over the pandemic (RV3). While all socio-demographic predictors in table 1 are included in the model, individual self-reported COVID-19 vaccine status is also included to further control for possible confounding on baseline attitudes when asking about change.^33^

### Model selection

Stepwise model selection using the Akaike Information Criterion (AIC) was performed to identify an appropriate form of each logistic regression model used in the analysis outlined above with interaction terms between survey year and socio-demographic variable permitted to explore directional changes in vaccine refusal within socio-demographic groups over time. Although stepwise AIC selection does not guarantee the optimal model, it provides a systematic way to balance model complexity and explanatory power, aiming for a model that better represents the data than simpler alternatives. This approach ensures that the final model captures relevant relationships between socio-demographic factors and vaccine refusal while adjusting for shifts in attitudes over time, minimising the risk of overfitting. The models with the lowest AIC are reported in the main text (see SM online supplemental tables 2-7).

### Patient and public involvement

No patients or members or the public were involved in setting the research question or the outcome measures, nor were they involved in developing plans for the design or implementation of the study.

All statistical analyses were performed in R.

## Results

### Changes in national and regional refusal over time

**Figure 1:**
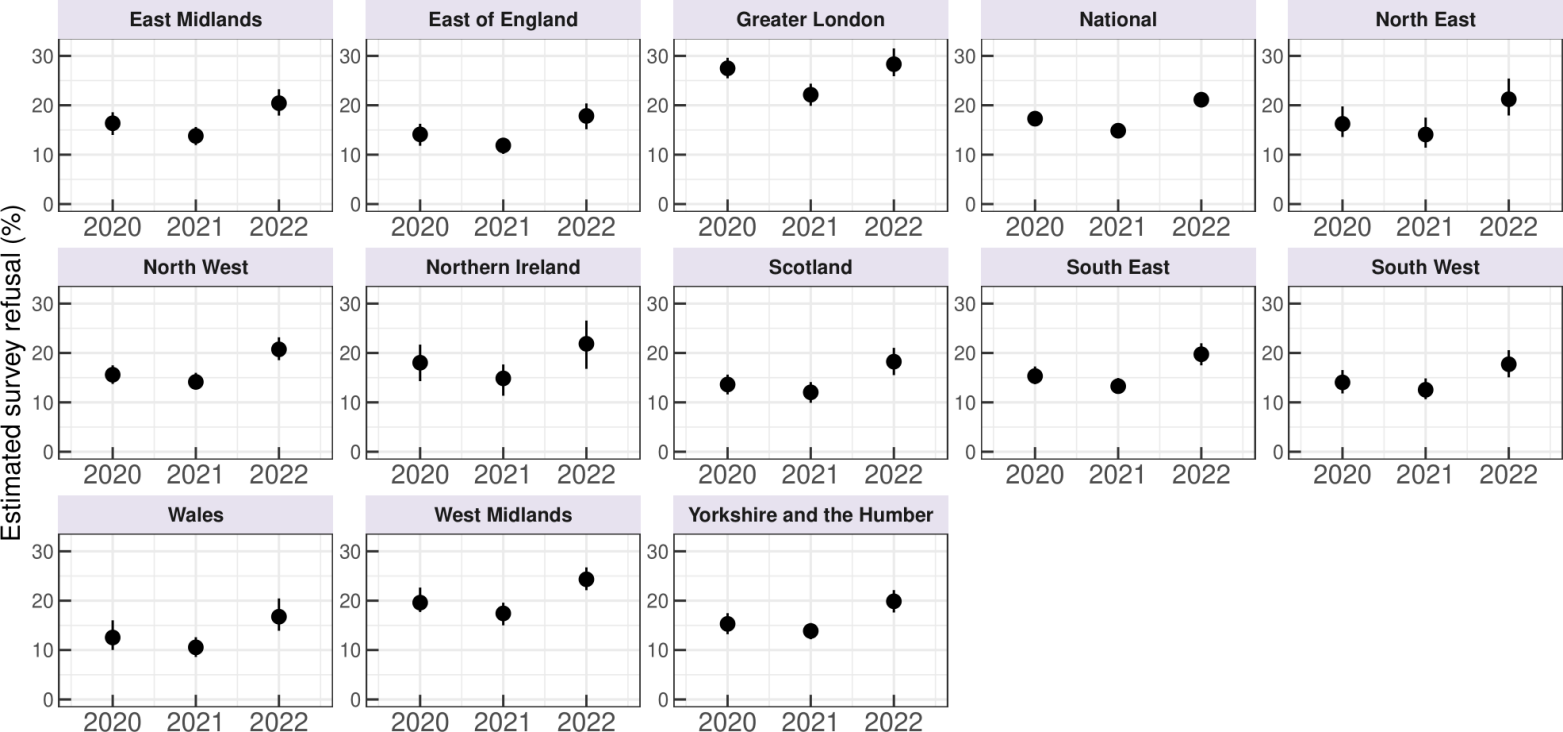
Temporal trends in vaccine refusal across the UK reveal increasing rates of refusal to any vaccine administered to children between 2020 and 2022. Estimated percentage of parents in the UK at the national level and each Nomenclature of Territorial Unit for Statistics level with 95% bootstrapped CIs who indicate that there is at least one immunisation they have not, or would not, allow their children to have (RV1).

**Figure 2:**
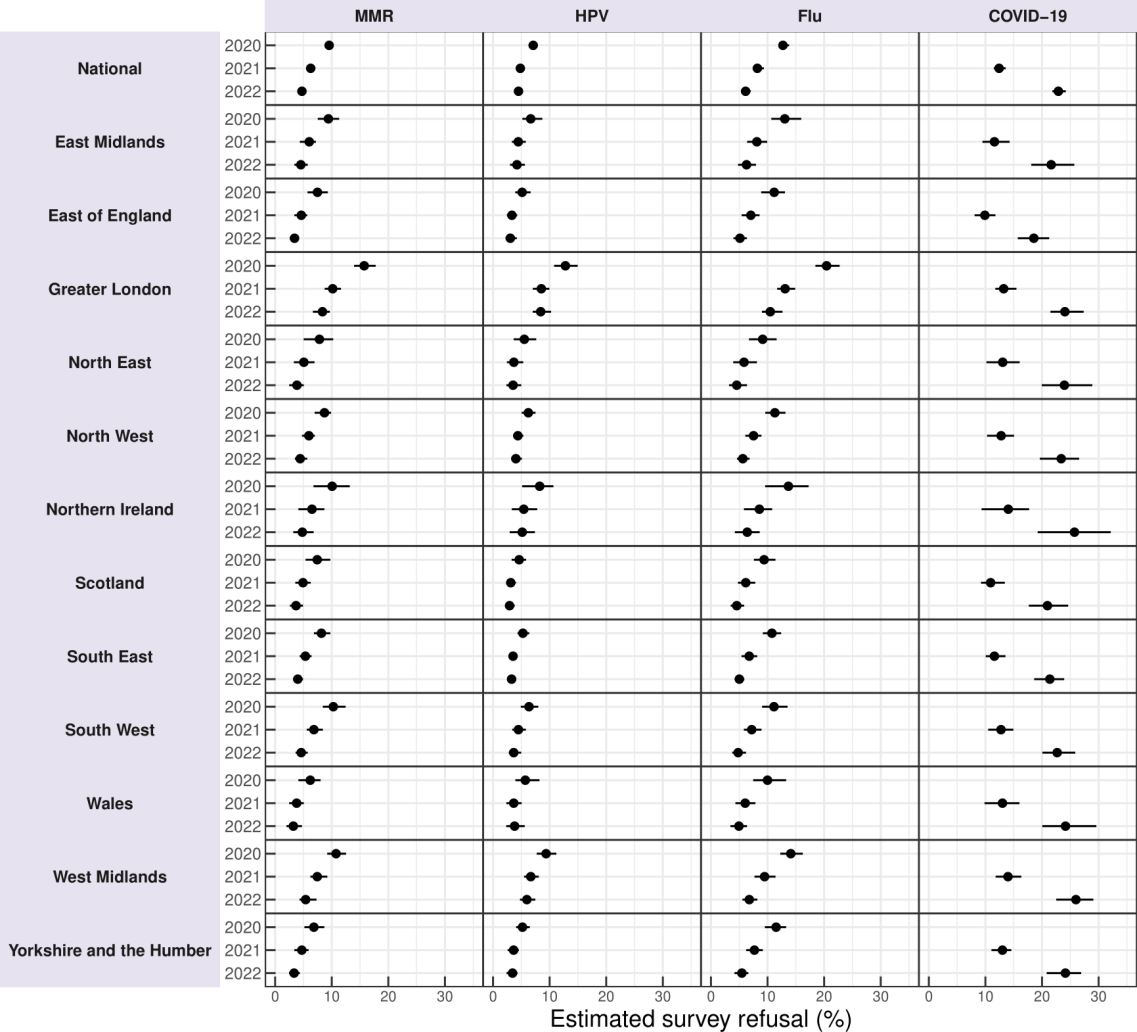
National and regional concern with MMR, HPV and influenza vaccines has decreased between 2020 and 2022, with concerns increasing markedly for the COVID-19 vaccines over the same period. Estimated percentage of parents in the UK at the national level with 95% bootstrapped CIs and each Nomenclature of Territorial Unit for Statistics level who indicate concerns about a specific vaccine (RV2).

Nationally, the reweighted predicted percentages of parents/carers refusing childhood vaccines were 17.3% (95% CI 16.4 to 18.5) in 2020, 14.8% (95% CI 13.7 to 15.7, p<0.001 compared with 2020) in 2021 and 21.1% (95% CI 19.8 to 22.5, p<0.001 compared with 2020) in 2022. A similar trend was observed across most regions (see figure 1), with a decline in reported refusal from 2020 to 2021, followed by an increase in 2022. Across all three surveys, the likelihood of vaccine refusal was consistently highest in Greater London. Sensitivity analyses yielded the same temporal trends (see online supplemental table 8); though excluding ‘don't know’ responses generally increased the percentage of respondents reporting vaccine refusal by 2 to 5 percentage points compared with when “don't know” responses were recoded as ‘no’. While refusal fell slightly in all regions of the UK in 2021 compared with 2020 before increasing to values in excess of those in 2022, concerns about specific vaccines reveal a contrasting picture (figure 2 and online supplemental table 9). Nationally, the proportion of parents/carers expressing concerns about the MMR and influenza vaccines declined from 2020 to 2021 (MMR: 9.6 to 6.3%, p<0.001; influenza: 12.6 to 8.2%, p<0.001) and again from 2021 to 2022 (MMR: 6.3 to 4.6%, p<0.001; influenza: 8.2 to 6.1%, p<0.001). This decreasing trend at the national level for these two vaccines was also observed consistently across all regions. For the HPV vaccine, a significant national decrease in reported concerns occurred from 2020 to 2021 (7.1 to 4.8%, p<0.001). There was no significant difference in reported concerns for the HPV vaccine between 2021 (4.8%) and 2022 (4.5%). Across all three of these vaccines, levels of concern were again highest in Greater London across all survey years (figure 2). For the COVID-19 vaccine, however, the number of parents who reported concerns rose significantly between 2021 and 2022 at the national level (12.4 to 23.0%, p<0.001) as well as for all regions.

### Socio-demographic determinants of vaccine refusal over time

**Figure 3:**
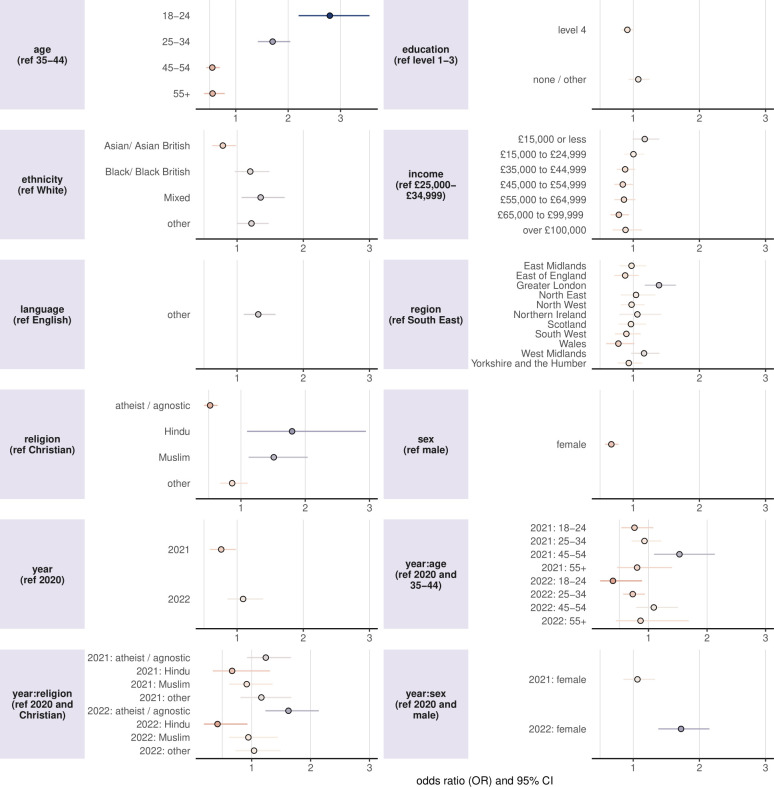
Socio-demographic determinants of vaccine refusal reveal an increase in vaccine refusal among females in 2022 compared with 2020 as well as higher odds of refusal among those in Greater London as well as Hindu and Muslim respondents. The results of the logistic regression of the impact of socio-demographic factors on the odds of respondents reporting that there is a vaccine they have or would refuse for their child(ren) (RV1). ORs larger than one indicate higher odds of refusal compared with the reference category. Interaction effects are denoted with a colon ‘:’. The response category ‘do not wish to answer’—while included in the regression—has been removed from the figure to improve figure visual clarity but can be found in online supplemental table 10.

The results of the logistic regression exploring the social, economic and demographic determinants of vaccine refusal (RV1) are shown in figure 3 (OR values are provided in online supplemental table 10 and OR for the sensitivity recoding are provided in online supplemental table 11). An OR larger than one indicates an association with higher refusal. Several socio-demographic factors are associated with vaccine refusal.

Female respondents were considerably less likely to report refusal than male respondents throughout the three survey years (OR 0.67, 95% CI 0.58 to 0.78), though this gap appears to have narrowed since 2020, with a positive interaction term between survey year and sex for 2022 (1.72, 1.38 to 2.15).

Compared with the reference age group (35–44 years), younger age groups were more likely to report vaccine refusal (18–24 years: 2.79, 2.20 to 3.55; 25–34 years: 1.70, 1.42 to 2.04), while older age groups were less likely to do so (45–54: 0.55, 0.44 to 0.70; 55 and over: 0.56, 0.39 to 0.79). Interaction terms with the survey year suggest these patterns shifted slightly over time. Among younger respondents, the odds of refusal decreased between 2020 and 2022 relative to the reference age group (18–24 years: 0.39 (0.17 to 0.89), 25–34 years: 0.73 (0.57 to 0.94)). A temporary increase in refusal was observed among 45–54-year-olds in 2021 (1.53, 1.10 to 2.13), with no significant change thereafter.

Hindu and Muslim respondents were more likely to report refusal compared with Christians (1.78, 1.10 to 2.90 and 1.51, 1.12 to 2.04, respectively), with atheist/agnostic respondents having lower rates of refusal than Christian respondents (0.52, 0.43 to 0.64). However, significant interaction terms with survey year indicate that these associations have changed over time. Among atheist/agnostic respondents, the odds of refusal increased between 2020 and 2022 (1.61, 1.23 to 2.10), whereas no such increase was observed in the Christian reference group. Despite this, atheists/agnostics remained less likely to report refusal across all survey years. In contrast, Hindu parents became significantly less likely to report refusal in 2022 compared with 2020 (0.42, 0.19 to 0.93), with no corresponding change in the Christian reference group. No significant change over time was observed for Muslim respondents.

Respondents with Asian or Asian British ethnicities were found to have lower rates of refusal than White respondents (0.78, 0.63 to 0.98), while those reporting Black or Black British or Mixed ethnicities had marginally higher rates of refusal than White respondents (1.20, 0.97 to 1.49 and 1.36, 1.07 to 1.72, respectively).

Those reporting a first language other than English were more likely to report refusal than those with English as a first language (1.32, 1.10 to 1.58). Higher levels of education (level-4) are found to be slightly more predictive of lower rates of reported refusal than those with level 1–3 education levels (0.91, 0.82 to 1.01) and higher incomes were also associated with lower rates of refusal. Consistent with findings from regional trends in refusal in figure 1, refusal rates in 2021 are lower than 2020 while individuals living in Greater London reported higher rates of refusal than those in the baseline category (South East), with individuals in the West Midlands also reporting slightly higher refusal rates.

### Self-reported changes in attitude to childhood immunisations

**Figure 4:**
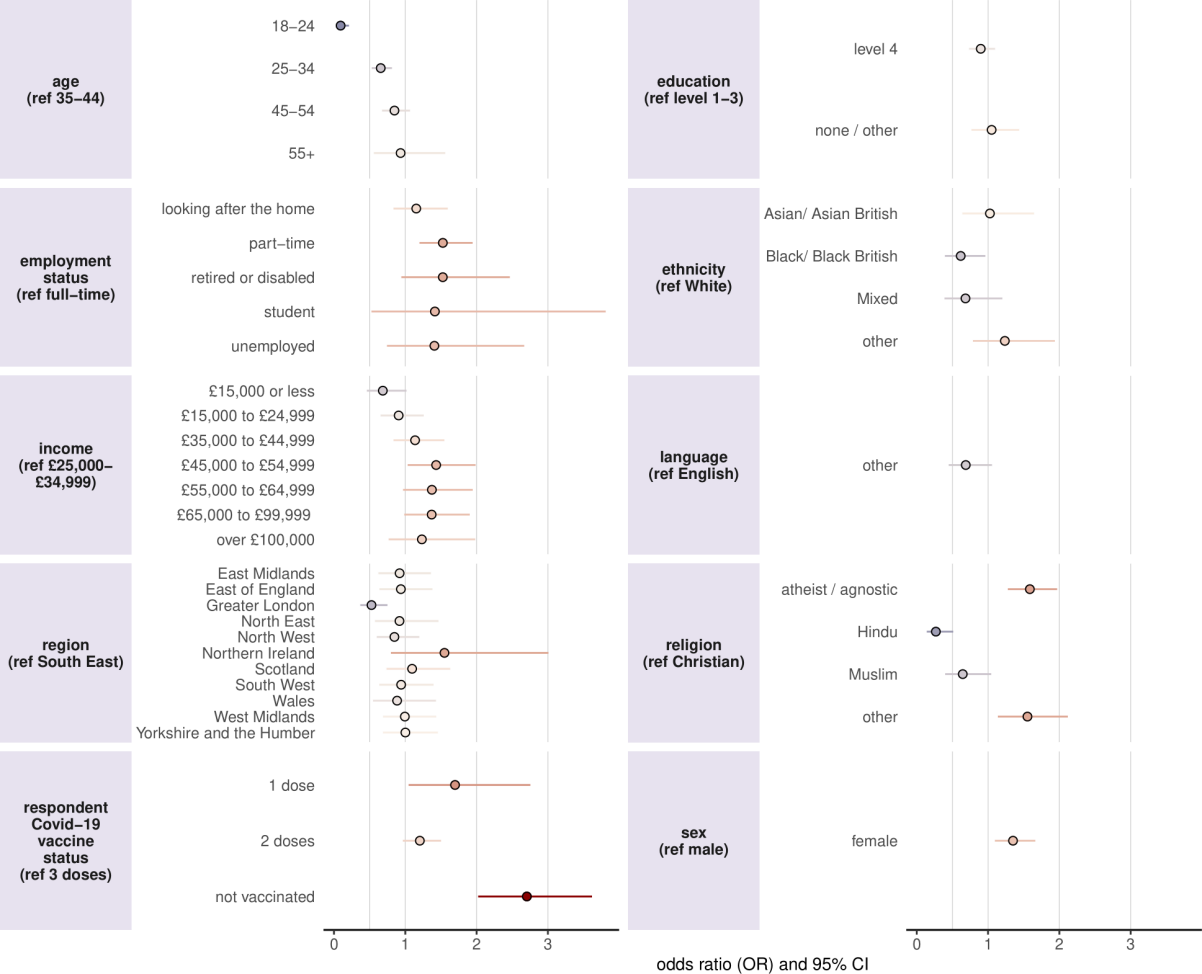
Socio-demographic determinants of changes in parental perceptions towards giving their children routine vaccines since the start of the COVID-19 pandemic. Parents who have not received at least three doses of a COVID-19 vaccine report being less positive in giving their children routine vaccines since the beginning of the COVID-19 pandemic compared with those who have received at least three doses. Younger age groups, those living in Greater London, Black/Black British respondents and Hindu respondents report a higher likelihood of being more positive about giving their children routine vaccines since the start of the pandemic, relative to their reference categories. ORs larger than one signify an increased negative sentiment towards childhood vaccinations, while ratios less than one signify an improved sentiment.

Overall, 84.3% of parents reported that their attitudes towards giving their children routine childhood vaccines had not changed (or they were unsure) since the beginning of the pandemic, while 9.2% reported that they had become more positive, and 6.5% had become less positive (unweighted values reported). The results of the ordinal logistic regression evaluating the determinants of the direction of change to changes in perceptions to routine childhood vaccines since the beginning of the pandemic (RV3) are shown in figure 4 and online supplemental table 12.

The strongest predictor of an increased negative perception towards routine childhood immunisations was found to be not being vaccinated with a COVID-19 vaccine (2.71, 2.02 to 3.62), with those without at least three doses also exhibiting increased negative perception (one dose: 1.68, 1.05 to 2.75; two doses: 1.20, 0.97 to 1.50). Working part-time (1.53, 1.20 to 1.94), being atheist/agnostic (1.59, 1.28 to 1.97), female (1.35, 1.10 to 1.66) and earning a middling salary were all also associated with an increase in negative perception compared with those working full-time, being Christian, males and a salary between £25 000 and £34 999, respectively. The strongest predictors of having become more positive about routine childhood vaccines were being aged 18–24 years (0.09, 0.04 to 0.21), being aged 25–34 (0.66, 0.53 to 0.81), living in Greater London (0.53, 0.37 to 0.75) and Hindu respondents (0.27, 0.14 to 0.51). Black/Black British respondents also report more positive attitudes to giving their children vaccines compared with White respondents (0.62, 0.40 to 0.96).

## Discussion

This study provides an in-depth examination of UK parental attitudes toward childhood vaccinations during the COVID-19 pandemic, capturing shifts in refusal rates and identifying key socio-demographic determinants of vaccine refusal and changing attitudes towards childhood vaccines. Our findings reveal a nuanced picture: while overall refusal rates for childhood vaccines initially decreased between 2020 and 2021, they rose again in 2022, surpassing 2020 levels. In contrast, specific concerns about MMR, HPV and influenza vaccines decreased consistently from 2020 to 2022, suggesting a divergence between general and specific vaccine attitudes, which was explained by increased concerns around COVID-19 vaccines for children. The decreased stated concerns for MMR, HPV and influenza vaccines are in contrast to observed coverage rates for these vaccines, all of which remain below pre-pandemic levels.^39^,^41^

Refusal rates were persistently higher in Greater London than in other UK regions. However, London’s 2022 survey responses suggest a relatively positive shift in vaccine attitudes, aligning with findings from the Office for National Statistics on decreasing COVID-19 vaccine hesitancy among Londoners.^42^ This regional trend underscores the importance of context-specific public health messaging and interventions.

Analysis of socio-demographic predictors of refusal (RV1) reveals that respondents who were younger, male, Hindu, Muslim, Black / Black British or spoke a first language other than English were more likely to report vaccine refusal than their respective reference groups across the 3 years of data. Conversely, higher education levels, higher income and Asian/Asian British respondents were less likely to report refusal. Interestingly, the two youngest age groups and Hindu and Black/British respondents reported a positive change in attitudes to childhood vaccines over the COVID-19 pandemic. By far the strongest predictor of an increased negative sentiment to childhood vaccines was respondents who had not received at least three doses of COVID-19 vaccines, suggesting a negative spillover effect among individuals who were not fully vaccinated against the COVID-19 vaccine. Overall, however, a small net increase in perceptions towards childhood vaccines was found, a result supported by data from an online survey carried out by the UK Health Security Agency in 2023 of 1000 parents and 1000 teenagers, which found no evidence that the COVID-19 vaccination programme had adversely impacted on attitudes to routine vaccines.^32^

### Study limitations

This study provides novel insights into shifts in parental confidence in routine childhood vaccines throughout the pandemic in the UK. The study does not explore reasons for past or anticipated refusals, including how these may depend on social or behavioural factors or child characteristics, which all play a part in decision-making processes. Thus, underlying causes for these trends cannot be inferred from the current data. Additionally, the introduction of additional vaccines (in this case, COVID-19) to a multiple-choice battery (as used in RV2) may induce survey bias into responses for other vaccines, which has the possibility of downwardly biasing estimates for concerns around other vaccines.

Although it is possible that the discrepancy could stem from another routine childhood vaccine not explicitly prompted in the survey, there was no indication of this from responses provided under the ‘other’ option in free text. While the question did specify ‘routine childhood vaccines’, some respondents may have interpreted this as including the COVID-19 vaccine, thus influencing their responses. It is further noted that there are many other child-level characteristics that may plausibly impact parental decision-making around vaccination that are not included in this study, such as child age or gender or the number of children that a parent has. The IPW approach deployed also assumes no additional unobserved confounders. Additionally, there are other unmeasured factors that may have driven differential rates in stated parental vaccination intent over the study period; for example, media information environment, survey timing and local disease outbreaks. Finally, our measures rely on self-reported attitudes and intentions, which are vulnerable to recall and social desirability biases and may not translate into realised uptake. As the analyses are observational and largely cross-sectional, causal inference is not possible.

### Conclusion

This study provides valuable insights into the evolving dynamics of parental and caregiver confidence in routine childhood vaccines in the UK during the COVID-19 pandemic. While vaccine refusal rates generally increased over the study period, the observed decrease in concerns about MMR, HPV and influenza vaccines suggests that primary concerns in 2022 were directed at COVID-19 vaccines for children. The marked negative shift in attitudes towards childhood vaccines among respondents who had not received at least three doses of a COVID-19 vaccine highlights a potential spillover effect of COVID-19 vaccine hesitancy onto routine childhood vaccines, and further research could focus on exploring hesitancy concerns in this cohort. The recent declines in MMR vaccine uptake across the UK do not appear to be linked to parental confidence in these vaccines, as indicated by this study’s findings. Further research is essential to understand the current drivers and barriers to childhood immunisations to address the decade-long slide in uptake.

## Supplementary material

10.1136/bmjph-2024-002536 online supplemental table 1

## Data Availability

Data are available upon reasonable request.
