# Supplementary material for: Changing parental perceptions to childhood immunisations during the COVID-19 pandemic in the UK: evidence from three cross-sectional surveys between 2020 and 2022
Source: BMJ Public Health. 2025 Nov 27;3(2):e002536. doi: 10.1136/bmjph-2024-002536 (PMC12666036; doi:10.1136/bmjph-2024-002536)
Supplement: online supplemental table 1 [file bmjph-3-2-s001.pdf]

Supplementary materials for Trends in self-reported parental refusal of childhood immunisations during the Covid-19 pandemic in the UK: evidence from three cross-sectional surveys between 2020 and 2022

Rosie Thistlethwayte[1] and Alexandre de Figueiredo[1,2†]

Table 1: number of responses by socioeconomic/ demographic group within each survey

	2020 (%)<br>N = 5288		2021 (%)<br>N = 4994		2022 (%)<br>N = 3988	
	N	%	N	%	N	%
**Sex**						
Female	2821	53.3	3074	61.6	2495	62.6
Male	2467	46.7	1924	38.5	1496	37.5
**Age group**						
18-24	444	8.4	435	8.7	34	0.9
25-34	1357	25.7	1309	26.2	1168	29.3
35-44	1829	34.6	1726	34.6	1730	43.4
45-54	1230	23.3	1141	22.8	934	23.4
55+	428	8.1	387	7.7	125	3.1
**Region**						
East Midlands	378	7.1	332	6.6	317	7.9
East of England	447	8.5	415	8.3	315	7.9
London	799	15.1	739	14.8	464	11.6
North East	220	4.2	209	4.2	189	4.7
North West	606	11.5	567	11.4	479	12.0
Northern Ireland	160	3.0	152	3.0	81	2.0
Scotland	404	7.6	381	7.6	310	7.8
South East	744	14.1	647	13.0	557	14.0
South West	359	6.8	373	7.5	309	7.7
Wales	202	3.8	224	4.5	181	4.5
West Midlands	506	9.6	530	10.6	410	10.3
Yorkshire and the Humber	463	8.8	425	8.5	376	9.4
**Education**						
Level 1-3	2144	40.5	2121	42.5	1642	41.2
Level 4	2495	47.2	2295	46.0	1913	48.0
None/ other	618	11.7	536	10.7	416	10.4
Do not wish to answer	31	0.6	44	0.9	19	0.5
**Work status**						
Full-time	3060	57.9	2785	55.8	2341	58.7
Part-time	1122	21.2	1130	22.6	940	23.6
Student	115	2.2	105	2.1	33	0.8
Unemployed	195	3.7	217	4.3	89	2.2
Looking after the home	545	10.3	502	10.1	421	10.6
Retired/ disabled	232	4.4	237	4.7	156	3.9
Do not wish to answer	19	0.4	22	0.4	11	0.3
**Religion**						
Atheist/ agnostic	1378	26.1	1258	25.2	1190	29.8
Christian	2543	48.1	2381	47.7	1748	43.8
Do not wish to answer	387	7.3	375	7.5	341	8.6
Hindu	100	1.9	118	2.4	70	1.8
Muslim	302	5.7	350	7.0	211	5.3
Other	578	10.9	516	10.3	431	10.8
**Ethnicity**						
Asian/ Asian British	394	7.5	469	9.4	289	7.2
Black/ Black British	181	3.4	188	3.8	164	4.1
Mixed	159	3.0	156	3.1	99	2.5
White	4122	78.0	3782	75.7	3211	80.5
Other	396	7.5	365	7.3	209	5.2
Do not wish to answer	36	0.7	38	0.8	19	0.5
**Language**						
English or Welsh	4746	89.8	4533	90.8	3740	93.8
Other	517	9.8	442	8.9	239	6.0
Do not wish to answer	25	0.5	19	0.4	9	0.2
**Income**						
Under ¬£15,000	525	9.9	487	9.8	315	7.9
¬£15,000 to ¬£24,999	859	16.2	777	15.6	513	12.9
¬£25,000 to ¬£34,999	881	16.7	921	18.4	666	16.7
¬£35,000 to ¬£44,999	807	15.3	761	15.2	626	15.7
¬£45,000 to ¬£54,999	677	12.8	638	12.8	544	13.6
¬£55,000 to ¬£64,999	455	8.6	430	8.6	429	10.8
¬£65,000 to ¬£99,999	613	11.6	552	11.1	539	13.5
Over ¬£100,000	206	3.9	200	4.0	170	4.3
Do not wish to answer	265	5.0	232	4.6	189	4.7

Table 2: Model comparison for RV1: “is there a vaccine you have or would refuse for your child?”

Model	Akaike Information Criterion (AIC)	Bayesian Information Criterion (BIC)
**All variables, no interactions**<br>Y ~ Year + Age + Sex + Income + Region + WorkStatus + Ethnicity + Education + Religion + Language	12,701	13,064
**Stepwise AIC final model**<br>Y ~ Age*Year + Sex*Year + Region + Income + Education + Ethnicity + Religion*Year + Language	12,656	13,125

Table 3: Model comparison for RV2: concerns around the MMR vaccine

Model	Akaike Information Criterion (AIC)	Bayesian Information Criterion (BIC)
**All variables, no interactions**<br>Y ~ Year + Age + Sex + Income + Region + WorkStatus + Ethnicity + Education + Religion + Language	6,983	7,346
**Stepwise AIC final model**<br>Y ~ Year + Age + Sex + Region + Income + Ethnicity + Religion + Language	6,977	7,272

Table 4: Model comparison for RV2: concerns around the influenza vaccine

Model	Akaike Information Criterion (AIC)	Bayesian Information Criterion (BIC)
**All variables, no interactions**<br>Y ~ Year + Age + Sex + Income + Region + WorkStatus + Ethnicity + Education + Religion + Language	8,371	8,743
**Stepwise AIC final model**<br>Y ~ Year + Age + Sex + Region + Education + Ethnicity + Religion + Language	8,364	8,621

Table 5: Model comparison for RV2: concerns around the HPV vaccine

Model	Akaike Information Criterion (AIC)	Bayesian Information Criterion (BIC)
**All variables, no interactions**<br>Y ~ Year + Age + Sex + Income + Region + WorkStatus + Ethnicity + Education + Religion + Language	5,941	6,304
**Stepwise AIC final model**<br>Y ~ Year + Age + Sex + Region + Income + Ethnicity + Religion + Language	5,924	6,203

Table 6: Model comparison for RV2: concerns around the Covid-19 vaccine

Model	Akaike Information Criterion (AIC)	Bayesian Information Criterion (BIC)
**All variables, no interactions**<br>Y ~ Year + Age + Sex + Income + Region + WorkStatus + Ethnicity + Education + Religion + Language	7,913	8,276
**Stepwise AIC final model**<br>Y ~ Education + WorkStatus + Ethnicity	7.890	8,117

Table 7: Model comparison for RV3 of “Since the beginning of the COVID-19 pandemic, how have your attitudes towards routine childhood vaccines changed?”

Model	Akaike Information Criterion (AIC)	Bayesian Information Criterion (BIC)
**All variables, no interactions**<br>Y ~ Year + CovidVaccinationStatus + Age + Sex + Income + Region + WorkStatus + Ethnicity + Education + Religion + Language	4,077	4,339
**Stepwise AIC final model**<br>Y ~ CovidVaccinationStatus + Age + Sex + Income + Region + WorkStatus + Religion + Language	4,075	4,325

Table 8: Predicted percentages of vaccine refusal ("is there a vaccine you have or would refuse for your child?") for the main analysis ("don't know" recoded to "no") and a sensitivity analysis that removes "don't know" responses.

Region	Model predicted percentage and 95% CI (2020)		Model predicted percentage and 95% CI (2021)		2022 Predicted probability (95% CI)	
	main analysis	sensitivity	main analysis	sensitivity	main analysis	sensitivity
National	17.3 (16.4, 18.5)	19.6 (18.5, 20.6)	14.8 (13.7, 15.7)	17.1 (15.9, 18.3)	21.1 (19.8, 22.5)	24.1 (22.5, 25.6)
East Midlands	16.4 (14.0, 18.6)	18.4 (15.0, 21.0)	13.8 (11.9, 15.6)	15.7 (13.3, 17.9)	20.4 (17.9, 23.2)	23.5 (20.1, 26.5)
East of England	14.1 (11.8, 16.2)	15.8 (13.1, 18.2)	11.9 (10.1, 13.3)	13.8 (11.6, 16.3)	17.8 (15.1, 20.4)	20.6 (17.4, 23.7)
Greater London	27.5 (25.5, 29.6)	31.6 (29.1, 34.1)	22.1 (19.9, 24.4)	26.0 (23.7, 29.0)	28.3 (25.9, 31.5)	32.0 (28.4, 35.6)
North East	16.2 (13.6, 19.8)	17.6 (15.0, 20.6)	14.1 (11.4, 17.5)	15.5 (12.7, 18.8)	21.2 (17.9, 25.4)	24.1 (19.9, 28.5)
North West	15.6 (13.7, 17.5)	17.9 (16.1, 20.0)	14.1 (12.6, 16.0)	16.4 (14.5, 18.8)	20.7 (18.5, 23.2)	23.7 (20.9, 26.1)
Northern Ireland	18.0 (14.3, 21.7)	20.1 (16.3, 24.0)	14.9 (11.3, 17.7)	16.6 (13.6, 20.5)	21.9 (16.8, 26.6)	24.2 (19.8, 29.2)
Scotland	13.6 (11.6, 15.6)	15.5 (13.6, 17.9)	12.0 (9.9, 14.1)	13.6 (11.6, 15.6)	18.2 (15.5, 21.0)	21.1 (18.2, 24.5)
South East	15.3 (13.7, 17.2)	16.9 (15.0, 19.1)	13.3 (11.9, 14.9)	15.1 (13.1, 16.8)	19.7 (17.5, 22.0)	22.2 (19.5, 24.7)
South West	14.0 (11.8, 16.5)	15.8 (13.9, 18.4)	12.5 (10.6, 14.8)	14.3 (12.2, 16.6)	17.7 (15.1, 20.6)	20.2 (17.3, 22.6)
Wales	12.5 (10.0, 16.0)	14.0 (10.5, 16.8)	10.5 (8.6, 12.6)	12.4 (9.9, 15.4)	16.8 (13.9, 20.4)	19.3 (15.0, 23.2)
West Midlands	19.6 (17.7, 22.6)	23.0 (20.8, 25.8)	17.4 (15.0, 19.6)	20.6 (17.9, 22.9)	24.3 (22.1, 26.7)	28.3 (25.7, 31.4)
Yorkshire and The Humber	15.3 (13.2, 17.5)	17.4 (15.1, 20.0)	13.9 (12.2, 15.5)	15.5 (13.4, 17.5)	19.9 (17.6, 22.1)	22.7 (20.0, 25.4)

Table 9: predicted probability of having concerns about MMR, HPV, Flu or COVID-19 vaccines

Region	Year	MMR	HPV	Flu	Covid-19
**National**	2020	9.6 (8.8, 10.4)	7.1 (6.5, 7.9)	12.6 (11.7, 13.4)	NA
**National**	2021	6.3 (5.6, 7.0)	4.8 (4.3, 5.3)	8.2 (7.6, 9.0)	12.4 (11.5, 13.3)
**National**	2022	4.6 (3.7, 5.3)	4.5 (4.0, 5.2)	6.1 (5.2, 7.0)	23.0 (21.6, 24.5)
**East Midlands**	2020	9.4 (7.6, 12.0)	6.5 (5.3, 8.1)	13 (11.1, 15.2)	NA
**East Midlands**	2021	6.0 (4.8, 7.5)	4.4 (3.2, 5.8)	8.2 (6.8, 9.9)	11.5 (9.3, 13.6)
**East Midlands**	2022	4.4 (3.3, 5.7)	4.2 (3.2, 5.3)	6.3 (5.1, 7.8)	21.5 (17.7, 25)
**East of England**	2020	7.2 (5.8, 9.2)	5.2 (3.4, 6.8)	11.1 (9.1, 13.1)	NA
**East of England**	2021	4.5 (3.3, 5.8)	3.3 (2.2, 4.5)	7.1 (5.9, 8.6)	9.9 (8.2, 11.7)
**East of England**	2022	3.2 (2.4, 4.2)	3.1 (2.1, 4.0)	5.1 (4.0, 6.3)	18.6 (15.7, 21.1)
**Greater London**	2020	15.9 (14, 17.7)	12.9 (11.3, 15.3)	20.1 (18, 22)	NA
**Greater London**	2021	10.2 (8.9, 11.6)	8.6 (7.3, 10.3)	13.1 (11.7, 14.7)	13.3 (11.5, 15.2)
**Greater London**	2022	8.1 (6.4, 9.4)	8.6 (7.1, 10.4)	10.3 (8.7, 12.1)	24.3 (21.1, 27.5)
**North East**	2020	7.7 (5.5, 10.3)	5.3 (3.1, 7.8)	9.2 (6.7, 11.6)	NA
**North East**	2021	5.0 (3.5, 6.7)	3.5 (2.2, 5.0)	5.9 (4.4, 7.9)	13.1 (10.0, 16.6)
**North East**	2022	3.7 (2.5, 4.9)	3.3 (1.8, 4.8)	4.7 (3.2, 6.6)	24.1 (19.0, 29.1)
**North West**	2020	8.8 (7.3, 10.6)	6.1 (4.8, 7.7)	11.2 (9.8, 12.6)	NA
**North West**	2021	6.0 (4.9, 7.2)	4.3 (3.4, 5.3)	7.5 (6.5, 8.7)	12.7 (10.8, 14.6)
**North West**	2022	4.4 (3.1, 5.3)	4.0 (3.1, 5.2)	5.6 (4.5, 6.9)	23.4 (20.6, 27.1)
**Northern Ireland**	2020	10.4 (7, 13.2)	8.0 (5.5, 11.6)	13.8 (10.2, 17.3)	NA
**Northern Ireland**	2021	6.6 (4.3, 8.5)	5.3 (3.4, 7.6)	8.8 (6.5, 11.3)	13.5 (9.8, 16.6)
**Northern Ireland**	2022	4.8 (3.0, 6.5)	5.0 (3.1, 7.2)	6.6 (4.4, 8.6)	24.8 (18.5, 31.2)
**Scotland**	2020	7.3 (5.6, 9.1)	4.7 (3.5, 6.3)	9.5 (7.5, 11.7)	NA
**Scotland**	2021	4.8 (3.6, 6.3)	3.2 (2.4, 4.4)	6.3 (5.0, 7.7)	10.8 (9.0, 13.0)
**Scotland**	2022	3.6 (2.4, 4.9)	3.0 (2.1, 3.9)	4.7 (3.6, 5.9)	20.9 (17.5, 23.9)
**South East**	2020	8.2 (7.0, 9.4)	5.4 (4.3, 6.7)	10.7 (9.2, 12.5)	NA
**South East**	2021	5.3 (4.2, 6.4)	3.6 (2.8, 4.3)	6.9 (5.8, 8.0)	11.4 (9.7, 13.1)
**South East**	2022	3.9 (3.0, 4.6)	3.4 (2.7, 4.3)	5.0 (4.0, 6.1)	21.3 (19.1, 24.2)
**South West**	2020	10.3 (7.8, 12.8)	6.4 (5.1, 8.4)	10.8 (8.1, 13.1)	NA
**South West**	2021	6.9 (5.4, 8.6)	4.5 (3.4, 5.7)	7.0 (5.4, 8.9)	12.9 (10.6, 15.6)
**South West**	2022	4.5 (3.2, 5.9)	3.7 (2.9, 5.0)	4.6 (3.5, 6.2)	23.2 (19.7, 27.3)
**Wales**	2020	6.3 (4.4, 8.3)	5.8 (3.7, 8.6)	10.2 (7.3, 12.7)	NA
**Wales**	2021	3.9 (2.6, 5.4)	3.7 (2.5, 5.1)	6.2 (4.3, 7.9)	13.4 (10.7, 16.8)
**Wales**	2022	3.2 (2.0, 4.5)	3.9 (2.5, 5.8)	5.1 (3.3, 6.8)	24.8 (21.0, 30.2)
**West Midlands**	2020	10.8 (8.9, 12.5)	9.3 (7.6, 10.9)	13.9 (11.7, 15.8)	NA
**West Midlands**	2021	7.5 (6.2, 8.8)	6.6 (5.2, 7.8)	9.4 (8, 11.0)	14.0 (12.6, 16.0)
**West Midlands**	2022	5.3 (4.0, 6.6)	6.0 (4.7, 7.5)	6.7 (5.5, 8.2)	26.2 (23.1, 29.9)
**Yorkshire and the Humber**	2020	6.9 (5.4, 8.5)	5.3 (4.1, 6.8)	11.4 (9.1, 13.1)	NA
**Yorkshire and the Humber**	2021	4.8 (3.8, 6.0)	3.7 (2.7, 4.8)	7.7 (6.1, 9.2)	13.1 (10.8, 15.5)
**Yorkshire and the Humber**	2022	3.3 (2.4, 4.3)	3.5 (2.6, 4.5)	5.4 (4.3, 6.5)	24.4 (20.5, 28.0)

Table 10: Odds of vaccine refusal by socioeconomic/ demographic group (with ‘Don’t Know’ responses recoded to ‘No’

Variable Group	Variable	OR (95% CI)	*p*-value
Age group (Ref = 35-44)	18-24yrs	2.796 (2.199, 3.553)	0.000
Age group (Ref = 35-44)	25-34yrs	1.703 (1.421, 2.041)	0.000
Age group (Ref = 35-44)	45-54yrs	0.551 (0.436, 0.697)	0.000
Age group (Ref = 35-44)	55+yrs	0.556 (0.392, 0.788)	0.001
Year (Ref = 2020)	2021	0.760 (0.589, 0.981)	0.035
Year (Ref = 2020)	2022	1.091 (0.855, 1.393)	0.483
Sex (Ref = Male)	Female	0.670 (0.576, 0.778)	0.000
Region (Ref = South East)	East Midlands	0.975 (0.792, 1.200)	0.808
Region (Ref = South East)	East of England	0.879 (0.714, 1.081)	0.221
Region (Ref = South East)	Greater London	1.390 (1.174, 1.645)	0.000
Region (Ref = South East)	North East	1.042 (0.814, 1.335)	0.743
Region (Ref = South East)	North West	0.975 (0.813, 1.170)	0.787
Region (Ref = South East)	Northern Ireland	1.063 (0.791, 1.428)	0.686
Region (Ref = South East)	Scotland	0.966 (0.781, 1.195)	0.751
Region (Ref = South East)	South West	0.897 (0.724, 1.112)	0.320
Region (Ref = South East)	Wales	0.776 (0.592, 1.018)	0.067
Region (Ref = South East)	West Midlands	1.164 (0.970, 1.396)	0.103
Region (Ref = South East)	Yorkshire and the Humber	0.933 (0.766, 1.137)	0.492
Income (Ref = £25,000-£34,999)	£15,000 to £24,999	1.003 (0.861, 1.169)	0.965
Income (Ref = £25,000-£34,999)	35,000 to £44,000	0.878 (0.752, 1.026)	0.101
Income (Ref = £25,000-£34,999)	£45,000 to £54,999	0.843 (0.715, 0.993)	0.041
Income (Ref = £25,000-£34,999)	£55,000 to £64,999	0.860 (0.716, 1.034)	0.108
Income (Ref = £25,000-£34,999)	£65,000 to £99,999	0.783 (0.657, 0.934)	0.007
Income (Ref = £25,000-£34,999)	Do not wish to answer	0.784 (0.613, 1.003)	0.053
Income (Ref = £25,000-£34,999)	Over £100,000	0.884 (0.689, 1.134)	0.332
Income (Ref = £25,000-£34,999)	Under £15,000	1.174 (0.989, 1.395)	0.067
Education (Ref = Level 1-3)	Do not wish to answer	0.751 (0.434, 1.300)	0.307
Education (Ref = Level 1-3)	Level 4	0.912 (0.823, 1.010)	0.078
Education (Ref = Level 1-3)	None/ other	1.077 (0.930, 1.246)	0.324
Ethnicity (Ref = White)	Asian/ Asian British	0.785 (0.626, 0.984)	0.036
Ethnicity (Ref = White)	Black/ Black British	1.199 (0.965, 1.489)	0.101
Ethnicity (Ref = White)	Do not wish to answer	0.960 (0.540, 1.708)	0.891
Ethnicity (Ref = White)	Mixed	1.356 (1.069, 1.720)	0.012
Ethnicity (Ref = White)	Other	1.217 (1.003, 1.478)	0.047
Religion (Ref = Christian)	Atheist/ agnostic	0.520 (0.425, 0.637)	0.000
Religion (Ref = Christian)	Do not wish to answer	1.018 (0.772, 1.343)	0.898
Religion (Ref = Christian)	Hindu	1.795 (1.095, 2.944)	0.020
Religion (Ref = Christian)	Muslim	1.510 (1.119, 2.039)	0.007
Religion (Ref = Christian)	Other	0.863 (0.675, 1.105)	0.242
First Language (Ref = English)	Do not wish to answer	1.941 (1.029, 3.662)	0.040
First Language (Ref = English)	Other	1.320 (1.101, 1.582)	0.003
Interaction: Year*Age	2021: 18-24yrs	0.760 (0.535, 1.081)	0.127
Interaction: Year*Age	2021: 25-34yrs	0.931 (0.711, 1.219)	0.604
Interaction: Year*Age	2021: 45-54yrs	1.529 (1.095, 2.133)	0.013
Interaction: Year*Age	2021: 55+yrs	0.806 (0.463, 1.401)	0.443
Interaction: Year*Age	2022: 18-24yrs	0.392 (0.173, 0.890)	0.025
Interaction: Year*Age	2022: 25-34yrs	0.730 (0.568, 0.940)	0.015
Interaction: Year*Age	2022: 45-54yrs	1.090 (0.790, 1.504)	0.600
Interaction: Year*Age	2022: 55+yrs	0.864 (0.443, 1.686)	0.668

Interaction: Year*Sex	2021: Female	1.064 (0.852, 1.329)	0.584
Interaction: Year*Sex	2022: Female	1.723 (1.379, 2.152)	0.000
Interaction: Year*Religion	2021: Atheist/ agnostic	1.239 (0.921, 1.667)	0.156
Interaction: Year*Religion	2022: Atheist/ agnostic	1.624 (1.233, 2.140)	0.001
Interaction: Year*Religion	2021: Do not wish to answer	1.097 (0.735, 1.638)	0.650
Interaction: Year*Religion	2022: Do not wish to answer	0.783 (0.523, 1.174)	0.237
Interaction: Year*Religion	2021: Hindu	0.668 (0.340, 1.310)	0.240
Interaction: Year*Religion	2022: Hindu	0.420 (0.190, 0.928)	0.032
Interaction: Year*Religion	2021: Muslim	0.915 (0.619, 1.353)	0.658
Interaction: Year*Religion	2022: Muslim	0.944 (0.619, 1.440)	0.788
Interaction: Year*Religion	2021: Other	1.164 (0.810, 1.673)	0.412
Interaction: Year*Religion	2022: Other	1.040 (0.726, 1.489)	0.830

Table 11: Odds of vaccine refusal by socioeconomic/ demographic group (sensitivity analysis: 'don't know' responses excluded)

Variable Group	Variable	OR (95% CI)	*p*-value
Age group (Ref = 35-44)	18-24yrs	2.856 (2.221, 3.674)	0.000
Age group (Ref = 35-44)	25-34yrs	1.666 (1.384, 2.005)	0.000
Age group (Ref = 35-44)	45-54yrs	0.553 (0.436, 0.702)	0.000
Age group (Ref = 35-44)	55+yrs	0.542 (0.381, 0.771)	0.001
Year (Ref = 2020)	2021	0.760 (0.586, 0.987)	0.039
Year (Ref = 2020)	2022	1.065 (0.830, 1.366)	0.623
Sex (Ref = Male)	Female	0.619 (0.531, 0.722)	0.000
Region (Ref = South East)	East Midlands	0.983 (0.795, 1.215)	0.873
Region (Ref = South East)	East of England	0.874 (0.708, 1.080)	0.212
Region (Ref = South East)	Greater London	1.435 (1.207, 1.707)	0.000
Region (Ref = South East)	North East	1.010 (0.784, 1.300)	0.941
Region (Ref = South East)	North West	0.999 (0.830, 1.203)	0.993
Region (Ref = South East)	Northern Ireland	1.092 (0.808, 1.476)	0.568
Region (Ref = South East)	Scotland	0.956 (0.770, 1.187)	0.684
Region (Ref = South East)	South West	0.894 (0.719, 1.113)	0.317
Region (Ref = South East)	Wales	0.783 (0.594, 1.032)	0.082
Region (Ref = South East)	West Midlands	1.209 (1.003, 1.457)	0.046
Region (Ref = South East)	Yorkshire and the Humber	0.927 (0.758, 1.133)	0.459
Income (Ref = £25,000-£34,999)	£15,000 to £24,999	1.024 (0.875, 1.197)	0.771
Income (Ref = £25,000-£34,999)	35,000 to £44,000	0.855 (0.729, 1.002)	0.053
Income (Ref = £25,000-£34,999)	£45,000 to £54,999	0.827 (0.699, 0.979)	0.027
Income (Ref = £25,000-£34,999)	£55,000 to £64,999	0.830 (0.688, 1.001)	0.051
Income (Ref = £25,000-£34,999)	£65,000 to £99,999	0.749 (0.626, 0.896)	0.002
Income (Ref = £25,000-£34,999)	Do not wish to answer	0.858 (0.665, 1.105)	0.235
Income (Ref = £25,000-£34,999)	Over £100,000	0.837 (0.649, 1.079)	0.170
Income (Ref = £25,000-£34,999)	Under £15,000	1.286 (1.076, 1.537)	0.006
Education (Ref = Level 1-3)	Do not wish to answer	0.834 (0.453, 1.536)	0.560
Education (Ref = Level 1-3)	Level 4	0.732 (0.398, 1.349)	0.318
Education (Ref = Level 1-3)	None/ other	0.928 (0.499, 1.726)	0.814
Ethnicity (Ref = White)	Asian/ Asian British	0.828 (0.656, 1.044)	0.111
Ethnicity (Ref = White)	Black/ Black British	1.285 (1.026, 1.609)	0.029
Ethnicity (Ref = White)	Do not wish to answer	1.085 (0.586, 2.010)	0.796
Ethnicity (Ref = White)	Mixed	1.390 (1.086, 1.780)	0.009
Ethnicity (Ref = White)	Other	1.350 (1.105, 1.650)	0.003
Religion (Ref = Christian)	Atheist/ agnostic	0.498 (0.406, 0.612)	0.000
Religion (Ref = Christian)	Do not wish to answer	1.072 (0.804, 1.430)	0.634
Religion (Ref = Christian)	Hindu	1.774 (1.061, 2.966)	0.029
Religion (Ref = Christian)	Muslim	1.516 (1.106, 2.078)	0.010
Religion (Ref = Christian)	Other	0.844 (0.655, 1.087)	0.188
First Language (Ref = English)	Do not wish to answer	2.655 (1.287, 5.477)	0.008
First Language (Ref = English)	Other	1.358 (1.125, 1.639)	0.001
Interaction: Year*Age	2021: 18-24yrs	0.737 (0.510, 1.064)	0.103
Interaction: Year*Age	2021: 25-34yrs	0.965 (0.732, 1.271)	0.799
Interaction: Year*Age	2021: 45-54yrs	1.445 (1.031, 2.026)	0.032
Interaction: Year*Age	2021: 55+yrs	0.769 (0.440, 1.344)	0.357
Interaction: Year*Age	2022: 18-24yrs	0.358 (0.155, 0.831)	0.017
Interaction: Year*Age	2022: 25-34yrs	0.767 (0.593, 0.994)	0.045
Interaction: Year*Age	2022: 45-54yrs	1.060 (0.764, 1.469)	0.728

Interaction: Year*Age	2022: 55+yrs	0.808 (0.411, 1.585)	0.535
Interaction: Year*Sex	2021: Female	1.090 (0.868, 1.370)	0.459
Interaction: Year*Sex	2022: Female	1.841 (1.465, 2.312)	0.000
Interaction: Year*Religion	2021: Atheist/ agnostic	1.254 (0.929, 1.694)	0.139
Interaction: Year*Religion	2022: Atheist/ agnostic	1.679 (1.268, 2.223)	0.000
Interaction: Year*Religion	2021: Do not wish to answer	1.136 (0.750, 1.722)	0.547
Interaction: Year*Religion	2022: Do not wish to answer	0.814 (0.535, 1.238)	0.336
Interaction: Year*Religion	2021: Hindu	0.630 (0.315, 1.261)	0.192
Interaction: Year*Religion	2022: Hindu	0.388 (0.172, 0.873)	0.022
Interaction: Year*Religion	2021: Muslim	0.900 (0.599, 1.351)	0.610
Interaction: Year*Religion	2022: Muslim	0.930 (0.599, 1.443)	0.745
Interaction: Year*Religion	2021: Other	1.229 (0.848, 1.783)	0.276
Interaction: Year*Religion	2022: Other	1.082 (0.748, 1.565)	0.675

Table 12: results of ordinal logistic regression predicting change in attitudes towards giving their children routine vaccines

Variable group	Variable	OR (95% CI)	p
Parental COVID-19 vaccine status (Ref = 3 doses)	1 dose	1.697 (1.046, 2.754)	0.032
Parental COVID-19 vaccine status (Ref = 3 doses)	2 doses	1.204 (0.966, 1.501)	0.099
Parental COVID-19 vaccine status (Ref = 3 doses)	Not vaccinated	2.705 (2.022, 3.618)	0.000
Age group (Ref = 35-44yrs)	18-24yrs	0.093 (0.041, 0.210)	0.000
Age group (Ref = 35-44yrs)	25-34yrs	0.655 (0.529, 0.812)	0.000
Age group (Ref = 35-44yrs)	45-54yrs	0.848 (0.675, 1.066)	0.157
Age group (Ref = 35-44yrs)	55+yrs	0.934 (0.560, 1.559)	0.794
Sex (Ref = Male)	Female	1.350 (1.096, 1.662)	0.005
Income (Ref = £25,000 - £34,999)	£15,000 to £24,999	0.907 (0.654, 1.258)	0.558
Income (Ref = £25,000 - £34,999)	35,000 to £44,000	1.137 (0.835, 1.548)	0.415
Income (Ref = £25,000 - £34,999)	£45,000 to £54,999	1.432 (1.033, 1.985)	0.031
Income (Ref = £25,000 - £34,999)	£55,000 to £64,999	1.373 (0.969, 1.945)	0.075
Income (Ref = £25,000 - £34,999)	£65,000 to £99,999	1.370 (0.986, 1.903)	0.061
Income (Ref = £25,000 - £34,999)	Do not wish to answer	1.442 (0.902, 2.304)	0.126
Income (Ref = £25,000 - £34,999)	Over £100,000	1.232 (0.767, 1.981)	0.388
Income (Ref = £25,000 - £34,999)	Under £15,000	0.684 (0.461, 1.017)	0.060
Region (Ref = South East)	East Midlands	0.920 (0.623, 1.358)	0.675
Region (Ref = South East)	East of England	0.938 (0.638, 1.380)	0.745
Region (Ref = South East)	Greater London	0.526 (0.369, 0.750)	0.000
Region (Ref = South East)	North East	0.918 (0.576, 1.465)	0.720
Region (Ref = South East)	North West	0.847 (0.600, 1.196)	0.346
Region (Ref = South East)	Northern Ireland	1.549 (0.798, 3.007)	0.196
Region (Ref = South East)	Scotland	1.095 (0.737, 1.629)	0.653
Region (Ref = South East)	South West	0.942 (0.635, 1.395)	0.764
Region (Ref = South East)	Wales	0.885 (0.548, 1.429)	0.617
Region (Ref = South East)	West Midlands	0.993 (0.688, 1.434)	0.972
Region (Ref = South East)	Yorkshire and the Hu mber	1.001 (0.688, 1.456)	0.997
Working status (Ref = full-time)	Do not wish to answer	0.191 (0.039, 0.950)	0.043
Working status (Ref = full-time)	Looking after the hom e	1.153 (0.834, 1.594)	0.388
Working status (Ref = full-time)	Part-time	1.526 (1.199, 1.944)	0.001
Working status (Ref = full-time)	Retired/ disabled	1.526 (0.944, 2.465)	0.084
Working status (Ref = full-time)	Student	1.415 (0.525, 3.810)	0.492
Working status (Ref = full-time)	Unemployed	1.408 (0.743, 2.667)	0.294
Ethnicity (Ref = White)	Asian/ Asian British	1.026 (0.640, 1.644)	0.915
Ethnicity (Ref = White)	Black/ Black British	0.616 (0.395, 0.962)	0.033
Ethnicity (Ref = White)	Do not wish to answer	0.902 (0.211, 3.866)	0.890
Ethnicity (Ref = White)	Mixed	0.683 (0.389, 1.200)	0.185
Ethnicity (Ref = White)	Other	1.235 (0.788, 1.937)	0.357
Education (Ref = Level 1-3)	Do not wish to answer	1.713 (0.459, 6.396)	0.423
Education (Ref = Level 1-3)	Level 4	0.898 (0.734, 1.097)	0.292
Education (Ref = Level 1-3)	None/ other	1.050 (0.768, 1.436)	0.760
Religion (Ref = Christian)	Atheist/ agnostic	1.586 (1.278, 1.969)	0.000
Religion (Ref = Christian)	Do not wish to answer	1.204 (0.849, 1.707)	0.298
Religion (Ref = Christian)	Hindu	0.268 (0.140, 0.512)	0.000

Religion (Ref = Christian)	Muslim	0.645 (0.399, 1.044)	0.074
Religion (Ref = Christian)	Other	1.552 (1.138, 2.119)	0.006
First Language (Ref = English)	Do not wish to answer	0.734 (0.116, 4.650)	0.743
First Language (Ref = English)	Other	0.687 (0.448, 1.055)	0.086

Questionnaire (2020)

Your views on the coronavirus/COVID-19 pandemic

You are invited to participate in this survey being conducted by researchers at Imperial College London to investigate the UK's views towards the coronavirus/COVID-19 pandemic. Your participation is **entirely voluntary**, and all your responses are **completely anonymous**. We are not collecting any information which can be used to identify you, such as your name, e-mail address, or home address.

The survey will take between 5 and 10 minutes to complete.

If you wish, you can exit the survey at any time, and we will not record your responses. Once you have submitted your responses, you will not be able to withdraw from the survey. Your responses are highly valuable to us and will be used to understand how adults in the UK feel about the coronavirus/COVID-19 pandemic. If you have any questions about this study, please contact us at socialhealthsurvey2020@imperial.ac.uk.

If you wish to participate please click the "Continue" button below. By clicking "Continue" you are indicating that you agree to participate. You must be at least 18 years old and reside in the UK.

DEMAGE How old are you?

▼ 18 (1) ... 100 (83)

DEMREG Which UK region do you live in?

▼ East Midlands (1) ... Yorkshire and The Humber (11)

DEM We would like to ask you some questions about yourself.
DEMSEX I am

- Male (1)
- Female (2)
- Other (3)

DEMEDU What is the highest level of education you have completed? (Select the response that best applies)

- No academic qualifications (1)
- 0-4 GCSE, O-levels, or equivalents (2)
- 5+ GCSE, O-levels, 1 A level, or equivalents (3)
- Apprenticeship (4)
- 2+ A levels or equivalents (5)
- Undergraduate or postgraduate degree, or other professional qualification (6)
- Other (e.g. vocational, foreign qualifications) (7)
- Do not know (8)
- Do not wish to answer (9)

DEMWRK Which of the following best describes your work status 6 months ago?

- Working full-time (including self-employed) (1)
- Working part-time (including self-employed) (2)
- Unemployed (3)
- Student (4)
- Looking after the home (5)
- Retired (6)
- Unable to work (e.g. short- or long-term disability) (9)

- Do not wish to answer (8)

DEMREL Do you consider yourself

- Christian (1)
- Hindu (2)
- Muslim (3)
- Jewish (4)
- Buddhist (5)
- Atheist or agnostic (6)
- Other (7)
- Do not wish to answer (8)

DEMETH Which best describes your ethnicity? (select the response that best applies)

- White: English/Welsh/Scottish/Northern Irish/British (1)
- White: Irish (2)
- White: Other white background (3)
- White and Black Caribbean (4)
- White and Black African (5)
- White and Asian or White and Asian British (6)
- Black, African, Caribbean or Black British (12)
- Asian or Asian British: Indian (7)
- Asian or Asian British: Pakistani (8)
- Asian or Asian British: Chinese (10)
- Asian or Asian British: Other (11)
- Other (13)
- Do not wish to answer (14)

DEMLAN What is your first language?

- English or Welsh (1)
- Polish (2)
- Punjabi (3)
- Urdu (4)
- Bengali (5)
- Other (6)
- Do not wish to answer (7)

DEMINC What is your total household income in GBP (£) from all sources before tax?

- Under £15,000 (1)
- £15,000 to £24,999 (2)
- £25,000 to £34,999 (3)
- £35,000 to £44,999 (4)
- £45,000 to £54,999 (5)
- £55,000 to £64,999 (6)
- £65,000 to £99,999 (7)
- Over £100,000 (8)
- Do not wish to answer (9)

End of Bloc

VAC_DEC Are you currently responsible for decisions relating to the vaccination of children?

- Yes (1)
- No (2)

VAC_CHI_REFUSE Are there any immunisations you have not, or would not, allow children to have?

- Yes (1)
- No (2)
- Do not know (7)

VAC_CHI_WHICH Which vaccines, if any, do you have concerns about? (select all that apply)

- ☐ The measles, mumps, and rubella combined vaccine (MMR) (1)
- ☐ The seasonal influenza vaccine (2)
- ☐ The human papilloma virus vaccine (HPV) (4)
- ☐ Other, please specify (5) ____
- ☐ ⊗None of the above (8)

End of Block

Questionnaire (2021)

Your views on the coronavirus/COVID-19 pandemic

You are invited to participate in this survey being conducted by researchers at the London School of Hygiene and Tropical Medicine (LSHTM) to investigate the UK's views towards the coronavirus/COVID-19 pandemic.
Your participation is **entirely voluntary**, and all your responses are **completely anonymous**. We are not collecting any information which can be used to identify you, such as your name, e-mail address, or home address.

The survey should take between 7 and 10 minutes to complete.

If you wish, you can exit the survey at any time, and we will not record your responses. Once you have submitted your responses, you will not be able to withdraw from the survey. **Your responses are highly valuable to us and will be used to understand how adults in the UK feel about the coronavirus/COVID-19 pandemic.** All your anonymous responses will be made available for academic use to better understand the pandemic in the UK and its impact on beliefs and behaviours.

If you wish to participate please click the next button "->" below. By clicking next you are indicating that you agree to participate and are happy to for your anonymous data to be shared with other academic researchers. You must be at least 18 years old and reside in the UK.

DEM We will begin by asking you some questions about yourself

DEMAGENUM How old are you?

▼ 18 (1) ... 100 (83)

DEMREG Which UK region do you live in?

▼ East Midlands (1) ... Other (for example, Jersey, Guernsey, Isle of Man) (13)

DEMSEX I am

- ○ Male (1)
- ○ Female (2)
- ○ Other (3)

DEMEDU What is the highest level of education you have completed? (Select the response that best applies)

- No academic qualifications (1)
- 0-4 GCSE, O-levels, or equivalents (2)
- 5+ GCSE, O-levels, 1 A level, or equivalents (3)
- Apprenticeship (4)
- 2+ A levels or equivalents (5)
- Undergraduate or postgraduate degree, or other professional qualification (6)
- Other (e.g. vocational, foreign qualifications) (7)
- Do not know (8)
- Do not wish to answer (9)

DEMWRK Which of the following best describes your work status 6 months ago?

- Working full-time (including self-employed) (1)
- Working part-time (including self-employed) (2)
- Unemployed (3)
- Student (4)
- Looking after the home (5)
- Retired (6)
- Unable to work (including, for example, a short- or long-term disability) (9)
- Do not wish to answer (8)

DEMREL Do you consider yourself

- ○ Christian (1)
- ○ Hindu (2)
- ○ Muslim (3)
- ○ Jewish (4)
- ○ Buddhist (5)
- ○ Atheist or agnostic (6)
- ○ Other (7)
- ○ Do not wish to answer (8)

DEMETH Which best describes your ethnicity (select the response that best applies)

- ○ White: English/Welsh/Scottish/Northern Irish/British (1)
- ○ White: Irish (2)
- ○ White: Other white background (3)
- ○ White and Black Caribbean (4)
- ○ White and Black African (5)
- ○ White and Asian or White and Asian British (6)
- ○ Black, African, Caribbean or Black British (12)
- ○ Asian or Asian British: Indian (7)
- ○ Asian or Asian British: Pakistani (8)
- ○ Asian or Asian British: Chinese (10)
- ○ Asian or Asian British: Other (11)
- ○ Gypsy or Irish traveller (16)
- ○ Other (13)
- ○ Do not wish to answer (14)
- ○ Roma (15)

DEMLAN What is your main language

- English or Welsh (1)
- Polish (2)
- Punjabi (3)
- Urdu (4)
- Bengali (5)
- Other (6)
- Do not wish to answer (7)

DEMINC What is your total household income in GBP (£) from all sources before tax?

- Under £15,000 (1)
- £15,000 to £24,999 (2)
- £25,000 to £34,999 (3)
- £35,000 to £44,999 (4)
- £45,000 to £54,999 (5)
- £55,000 to £64,999 (6)
- £65,000 to £99,999 (7)
- Over £100,000 (8)
- Do not wish to answer (9)

Start of Block

VAC_DEC Are you **currently** responsible for decisions relating to the vaccination of children?

- Yes (1)
- No (2)

VAC_CHI_REFUSE Are there any immunisations you have not, or would not allow children to have?

- ◯ Yes (1)
- ◯ No (2)
- ◯ Do not know (3)

VAC_CHI_WHICH Which vaccines, if any, do you have concerns about (select all that apply)

- ▢ The measles, mumps, and rubella combined vaccine (MMR) (1)
- ▢ The seasonal influenza vaccine (2)
- ▢ The human papilloma virus vaccine (HPV) (3)
- ▢ The coronavirus (COVID-19) vaccines (4)
- ▢ Other, please specify (5) ____
- ▢ ⊗None of the above (6)

Questionnaire (2022)

Your views on the coronavirus/COVID-19 pandemic

You are invited to participate in this survey being conducted by researchers at the London School of Hygiene and Tropical Medicine (LSHTM) to investigate the UK's views towards the coronavirus/COVID-19 pandemic.

Your participation is **entirely voluntary**, and all your responses are **completely anonymous**. We are not collecting any information which can be used to identify you, such as your name, e-mail address, or home address.

The survey should take no longer than 5 minutes to complete.

If you wish, you can exit the survey at any time, and we will not record your responses. Once you have submitted your responses, you will not be able to withdraw from the survey. **Your responses are highly valuable to us and will be used to understand how adults in the UK feel about the coronavirus/COVID-19 pandemic.** All your anonymous responses will be made available for academic use to better understand the pandemic in the UK, but as a reminder, your responses will **always stay completely anonymous**.

If you wish to participate please click the next button "->" below. By clicking next you are indicating that you agree to participate and are happy to for your anonymous data to be shared with other academic researchers. You are also acknowledging that you are at least 18 years old and reside in the UK.

VAC_DEC Are you currently responsible for decisions relating to the vaccination of children?

- ○ Yes (1)
- ○ No (2)

SOCDEM We will begin by asking you some questions about yourself

AGE How old are you?

▼ 18 (1) ... 100 (83)

REG Which UK region do you live in?

▼ East Midlands (1) ... Other (for example, Jersey, Guernsey, Isle of Man) (13)

SEX I am

- ○ Male (1)
- ○ Female (2)
- ○ Other (3)

EDU What is the highest level of education you have completed? (Select the response that best applies)

- ○ No academic qualifications (1)
- ○ 0-4 GCSE, O-levels, or equivalents (2)
- ○ 5+ GCSE, O-levels, 1 A level, or equivalents (3)
- ○ Apprenticeship (4)
- ○ 2+ A levels or equivalents (5)
- ○ Undergraduate or postgraduate degree, or other professional qualification (6)
- ○ Other (e.g. vocational, foreign qualifications) (7)
- ○ Do not know (8)
- ○ Do not wish to answer (9)

EMP Which of the following best describes your work status 6 months ago?

- ○ Working full-time (including self-employed) (1)
- ○ Working part-time (including self-employed) (2)
- ○ Unemployed (3)
- ○ Student (4)
- ○ Looking after the home (5)
- ○ Retired (6)
- ○ Unable to work (including, for example, a short- or long-term disability) (9)
- ○ Do not wish to answer (8)

REL Do you consider yourself

- ○ Christian (1)
- ○ Hindu (2)
- ○ Muslim (3)
- ○ Jewish (4)
- ○ Buddhist (5)
- ○ Atheist or agnostic (6)
- ○ Other (7)
- ○ Do not wish to answer (8)

ETH Which best describes your ethnicity (select the response that best applies)

- ◯ White: English/Welsh/Scottish/Northern Irish/British (1)
- ◯ White: Irish (2)
- ◯ White: Other white background (3)
- ◯ White and Black Caribbean (4)
- ◯ White and Black African (5)
- ◯ White and Asian or White and Asian British (6)
- ◯ Black, African, Caribbean or Black British (12)
- ◯ Asian or Asian British: Indian (7)
- ◯ Asian or Asian British: Pakistani (8)
- ◯ Asian or Asian British: Chinese (10)
- ◯ Asian or Asian British: Other (11)
- ◯ Gypsy or Irish traveller (16)
- ◯ Roma (15)
- ◯ Other (13)
- ◯ Do not wish to answer (14)

LAN What is your main language

- English or Welsh (1)
- Polish (2)
- Punjabi (3)
- Urdu (4)
- Bengali (5)
- Other (6)
- Do not wish to answer (7)

INC What is your total **household** income in GBP (£) from all sources before tax?

- Under £15,000 (1)
- £15,000 to £24,999 (2)
- £25,000 to £34,999 (3)
- £35,000 to £44,999 (4)
- £45,000 to £54,999 (5)
- £55,000 to £64,999 (6)
- £65,000 to £99,999 (7)
- Over £100,000 (8)
- Do not wish to answer (9)

Start of Block: Childhood immunisations: non-COVID (1)

VAC_CHI_REFUSE Are there any immunisations you have not, or would not, allow children to have?

- Yes (1)
- No (2)
- Do not know (4)

VAC_CHI_WHICH Which vaccines, if any, do you have concerns about? (select all that apply)

- The measles, mumps, and rubella combined vaccine (MMR) (1)
- The seasonal influenza vaccine (2)
- The coronavirus (COVID-19) vaccines (6)
- The human papilloma virus vaccine (HPV) (3)
- Other, please specifiy (4) ____
- ⊗None of the above (5)

CHILD_CONF Since the beginning of the COVID-19 pandemic, has your attitude towards giving your child/children routine vaccines changed? (The Covid-19 vaccine is not considered a routine vaccination.)

- Yes (1)
- No (2)
- Do not know (3)

CHILD_CONF_HOW How have your attitudes towards other vaccines for your child/children changed?

- I feel much more positive about giving my child vaccines (1)
- I feel slightly more positive about giving my child vaccines (2)
- I feel slightly less positive about giving my child vaccines (3)
- I feel much less positive about giving my child vaccines (4)
